# Pancreatic insulinoma: Diagnosis and treatment of a rare tumour with misleading symptoms — A case report

**DOI:** 10.1016/j.ijscr.2023.108603

**Published:** 2023-08-01

**Authors:** Emmanuel Agbozo

**Affiliations:** Ghana Health Service, Ghana

**Keywords:** Insulinoma, Hypoglycaemia, Neuroendocrine tumour, Seizure, Case report

## Abstract

**Introduction:**

Insulinomas are an uncommon occurrence, with an annual incidence of approximately 4 per million. These functional pancreatic neuroendocrine tumours can present with a myriad of nonspecific symptoms leading to frequent misdiagnoses.

**Presentation of case:**

In this case report is presented a 55-year-old man who was misdiagnosed and managed for a seizure disorder with escalating antiepileptic treatments for 11 months. A thorough history after an attack was the main tool in solving the mystery of his refractory seizures, leading to the discovery of a pancreatic insulinoma. Biochemical tests revealed fasting hypoglycaemia and a relative hyperinsulinemia, and a distal pancreatic lesion measuring approximately 1.8 cm × 1.3 cm was detected on CT, MRI and endoscopic ultrasound. Successful laparoscopic pancreatic left resection led to complete resolution of symptoms and restoration of quality of life to pre-illness levels.

**Discussion and conclusion:**

Insulinomas have historically been difficult to diagnose because their symptoms mimic neurologic and psychiatric conditions. Patterns of symptom occurrence obtained from a carefully-taken history is the single most important tool in assessing patients with insulinomas, who usually present with unusual and refractory neuropsychiatric conditions.

## Highlights

• Insulinomas are often misdiagnosed as neuropsychiatric conditions
• A thorough history is the key primary tool in identifying potential insulinomas
• Relative, not absolute, elevation in insulin and C-peptide is critical in diagnosis
• Surgical resection restores QOL to pre-illness levels with minimal complications

## 1 Introduction

Insulinomas are notable for being the most common cause of endogenous hyperinsulinism and an uncommon occurrence, with an annual incidence of approximately 4 per million [1,2]. These functional pancreatic neuroendocrine tumours can present with a myriad of symptoms resulting from the neuroglycopenic and/or autonomic stress responses to hypoglycaemia. Due to the rare nature of insulinomas coupled with the relatively nonspecific nature of associated symptoms, most patients are left misdiagnosed and symptomatic for long periods of time. In a retrospective case series of 59 patients between 1951 and 1996 at the Cleveland Clinic Foundation, only 28 % of patients were diagnosed within one year of symptom onset. [3].

This report highlights a case of pancreatic insulinoma misdiagnosed as a seizure disorder to remind clinicians to carefully consider surgically-treatable metabolic causes like insulinoma in the evaluation of patients with unusual and refractory neurological symptoms.

This work has been reported in line with SCARE criteria [4].

## 2 Case report

### 2.1 Presentation

A male patient in his mid-fifties was seen following a sudden morning episode of generalized muscle weakness, slurred speech and diaphoresis with no confusion or loss of consciousness. His symptoms slowly abated after prompt administration of a sugary drink at home. He had a background medical history of hypertension and familial hypercholesterolemia for which he was being appropriately managed. He had a healthy lifestyle typically eating his last meal of the day between 3 pm and 5 pm followed by at least 45 min of moderate-intensity exercise before bedtime.

At presentation, physical examination was unremarkable. The patient was diagnosed of a resolved probable hypoglycaemic episode based on two out of three Whipple triad criteria. He had no history of diabetes mellitus and had never used exogenous insulin or other hypoglycaemic agents. Further history revealed he had been diagnosed with a generalized seizure disorder about 11 months prior, after experiencing recurrent episodes of disorientation. The episodes were usually in the early morning hours and could last several minutes, followed by spontaneous recovery and amnesia. There was no loss of consciousness, change in muscle tone or history of convulsions during these episodes. Several months prior to these notable episodes, he had also been seen a few mornings by his daughter staring blankly and appearing confused for a few minutes.

He had been seen at a neurology clinic where a sleep-deprived electroencephalogram and brain MRI were done. The EEG showed localized spike and wave activity in the left temporal hemispheric areas as well as paroxysmal generalized spike and wave discharges, and brain MRI revealed no gross pathology. A diagnosis of generalized seizure disorder was made and therapy with Levetiracetam 500 mg bd was initiated. Dose adjustments over several months did not seem to improve the occurrence of his episodes and Lacosamide 50 mg bd was added to his treatment regimen (later increased to 100 mg bd). His symptoms however continued to occur with increased frequency and duration compared to earlier episodes, despite maximal doses of anti-epileptic drugs.

The episodes of disorientation were presumed by the patient to be unrelated to the acute episode at presentation. However, based on the timing of the patient's symptoms coupled with his lifestyle of prolonged fasting and exercise, there was a high index of suspicion for recurrent hypoglycaemic episodes secondary to endogenous hyperinsulinism. A pancreatic insulinoma was the main diagnosis under consideration for investigation.

### 2.2 Investigations

A supervised fast was conducted and terminated after the patient had symptoms consistent with hypoglycaemia at a plasma glucose level of 3.6 mmol/l. Plasma insulin was 5.0μIU/ml (range: 2.6–24.9) and C-peptide, 1.89 ng/ml (range: 1.10–4.40). These normal and euglycemic values obtained represented an inappropriate elevation of insulin and C-peptide in the setting of hypoglycaemia (i.e., a relative and not an absolute hyperinsulinemia) – critical in the biochemical diagnosis of endogenous hyperinsulinism. Glycated haemoglobin was 4.97 %.

**Fig. 1:**
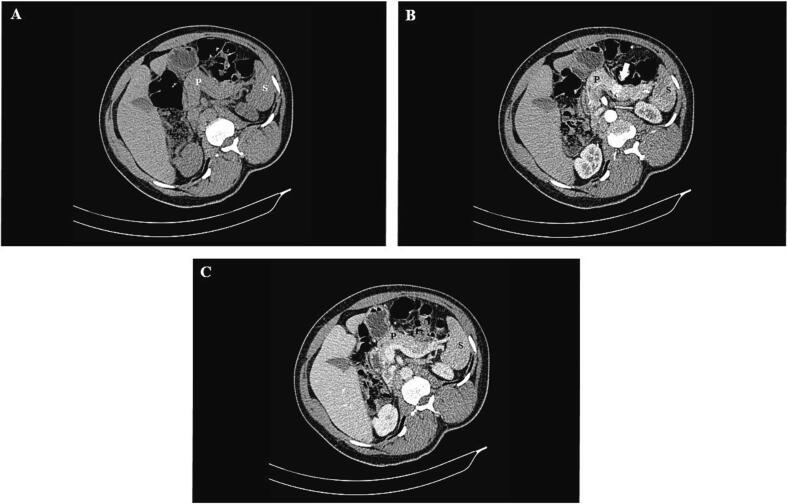
Multiphase abdominal CT scan showing (A) pre-contrast, (B) arterial, and (C) portal venous phases with a hypervascular lesion in the distal pancreas displaying more enhancement during the arterial phase (white arrow in image B). P – pancreas; S – spleen.

**Fig. 2:**
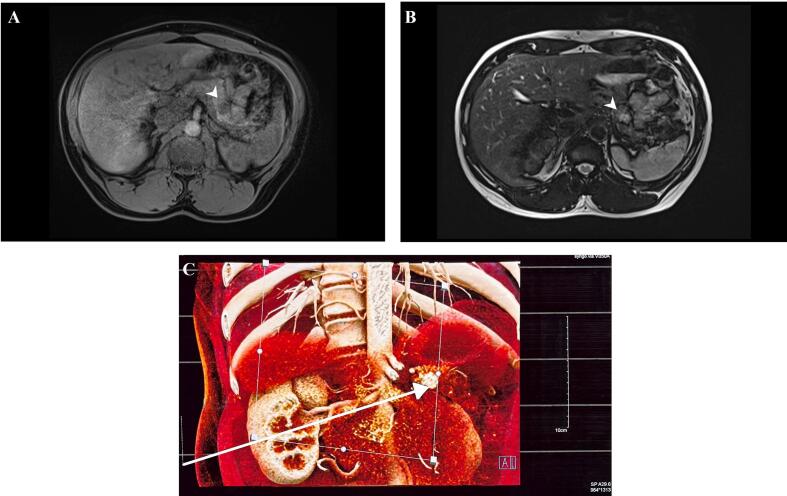
Abdominal MRI scan showing a pancreatic tumour (white arrowhead), (A) hypointense on T1 fat-suppressed sequence and (B) hyperintense on T2 fat-suppressed sequence – the classic imaging finding of pancreatic insulinomas. (C) 3D reconstruction of MRI sequences showing the insulinoma in the distal pancreas (white arrow).

A multiphase computed tomography scan of the abdomen showed a hypervascular lesion involving the distal pancreas which measured 1.8 cm × 1.3 cm and was only visible during the arterial phase (Fig. 1). A non-enhanced abdominal magnetic resonance imaging scan showed a well-defined T2 hyperintense rounded lesion, hypointense on T1 and with no restricted diffusion (Fig. 2). There were no regional lymph node enlargements and no lesions suspicious of intra-abdominal metastasis.

**Fig. 3:**
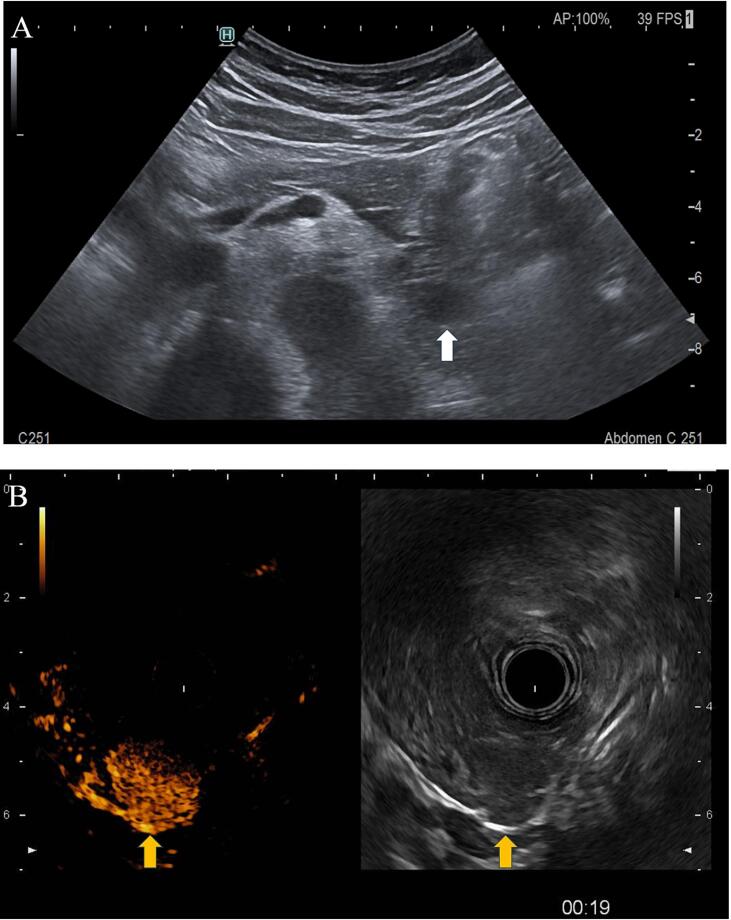
(A) Transabdominal ultrasound showing the insulinoma in the distal pancreas (white arrow). (B) Contrast-enhanced EUS showing insulinoma (yellow arrow). (For interpretation of the references to colour in this figure legend, the reader is referred to the web version of this article.)

Endoscopic ultrasound showed a weakly echogenic, sharply limited and polylobulated tumour measuring 1.6 cm × 1.4 cm in the tail region of the pancreas, about 0.1 cm from the main pancreatic duct. The lesion was strongly vascularized on colour-coded duplex sonography and showed early arterial perfusion on contrast-enhanced ultrasonography (Fig. 3).

### 2.3 Treatment

Preoperative EUS-guided fine needle tattooing (EUS-FNT) was done to improve tumour localization, followed three days later by successful laparoscopic pancreatic left resection with splenic preservation.

On the first postoperative day, effluent from a drain left in-situ showed the output contained pancreatic secretions confirmed by high amylase levels. This was managed conservatively with drain output regressing significantly over the course of a few days. The drain was removed on postoperative day 4 and patient was discharged on postoperative day 7.

### 2.4 Outcome and follow-up

**Fig. 4:**
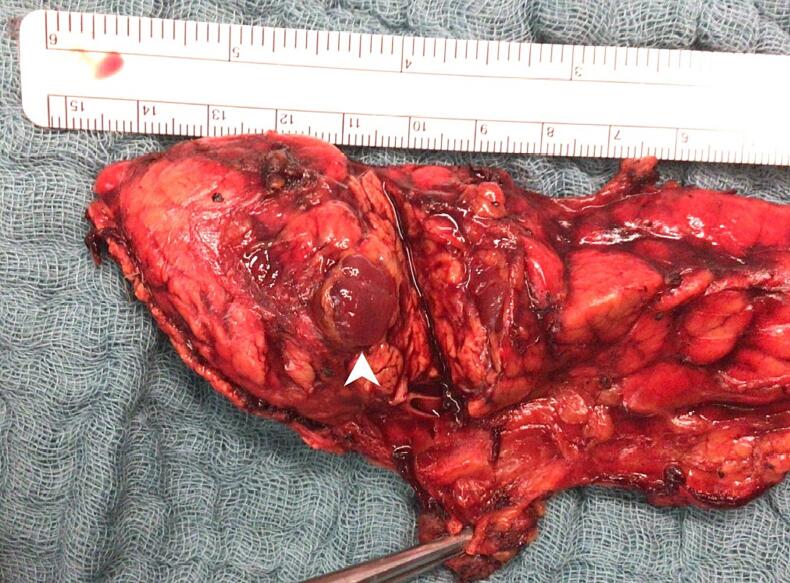
Resected pancreatic specimen showing the insulinoma (white arrowhead).

Histopathological analysis of the surgical specimen showed pancreatic parenchyma with a tumour up to 1.6 cm in size and tumour-free margins of <1 mm (Fig. 4). The tumour was predominantly trabecular in structure and morphologically compatible with a neuroendocrine tumour. Immunohistochemically, the cells expressed pancytokeratin, CD56, synaptophysin and chromogranin A. Insulin was also positive immunohistochemically. Ki67 was 4 % to 5 %. Findings confirmed complete resection of a benign pancreatic insulinoma.

The patient was evaluated 6 and 12-months post-surgery and reported being symptom-free over the period. Biochemical tests showed fasting glucose levels of 5.5 mmol/l with insulin of 4.3μIU/ml and C-peptide of 1.31 ng/ml. Glycated haemoglobin was now in the pre-diabetic range, at a level of 6.43 %, and the patient was given appropriate dietary and lifestyle counselling to improve glucose control.

## 3 Discussion

Insulinomas are functional pancreatic islet beta-cell tumours causing hypoglycaemia due to endogenous hyperinsulinism. >90 % of insulinomas are benign and it is associated with MEN1 in 6–7.6 % of patients [5]. The underlying pathophysiology in this condition is autonomous insulin secretion resulting in a failure of insulin secretion to fall to very low levels in response to hypoglycaemia [1], potentiating its hypoglycaemic effect. Hypoglycaemia is clearly documented by the Whipple's triad of (1) symptoms consistent with hypoglycaemia, (2) a low measured plasma glucose level and (3) resolution of symptoms after the plasma glucose level is raised [1,6].

The symptoms of hypoglycaemia are categorized into two major groups: neuroglycopenic symptoms resulting directly from CNS glucose deprivation, and autonomic symptoms from CNS-mediated sympathetic overdrive in response to neuroglycopenic stress [1,7,8]. Neuroglycopenic symptoms include lethargy, visual changes, confusion, amnesia, personality changes or abnormal behaviour, neurologic deficits, seizures, and in severe cases, coma and death [1,3,7]. Autonomic symptoms include palpitations, tremor, diaphoresis, anxiety, paraesthesia and hunger. The symptoms of hypoglycaemia are seen in varying combinations in patients with insulinoma, with neuroglycopenic symptoms usually being more predominant [8,9]. Due to the nonspecific nature of symptoms in patients with insulinoma, there is a significant delay in diagnosis, sometimes lasting decades [9]. The median time to diagnosis according to a series of 59 patients seen at the Cleveland Clinic was 24 months from symptom onset, with the majority of patients being diagnosed within 1 to 5 years, and only 28 % being diagnosed within the first year [3]. Due to the common occurrence of neuroglycopenic symptoms, neurologic and psychiatric misdiagnoses are common in this patient population [2,3,10].

An important key to arriving at a diagnosis in these patients presenting with unusual neurological symptoms is a carefully taken history seeking out the timing and pattern of symptom occurrence. Although there may be no noticeable pattern of occurrence in some patients, it is apparent that in a large number of patients, similar to this case report, symptoms tend to occur after a period of fasting or caloric deficit such as at night-time or in the early morning hours, following skipped or delayed meals, or after exercise [5,9,11].

The electrical activity of the brain, measured by EEG, is closely related to the metabolic state of brain cells [12]. Neuroglycopenia has been found to be associated with diffuse and symmetrical cortical EEG changes [13]. These changes tend to be predominant temporally and parieto-occipitally, with more severe hypoglycaemia causing 2-4 Hz bisynchronous bursts of activity frontotemporally: similar waves can be seen in absence and other generalized seizures, contributing to misdiagnosis [13,14].

The supervised 72-h fast is the gold standard for establishing the diagnosis of insulinoma. During this test, plasma measurements of glucose, insulin, C-peptide and proinsulin are made 6-hourly till glucose falls to or below 3.3 mmol/l, followed by 1–2 hourly measurements. The test is terminated when glucose falls below 2.5 mmol/l or the patient develops symptoms.

Following biochemical diagnosis of insulinoma, preoperative localization may be done. This involves non-invasive modalities such as transabdominal ultrasonography, CT and MRI which have often failed to localize these frequently small tumours which are <2 cm 90 % of the time; and invasive modalities such as angiography and endoscopic ultrasound [1,7,15]. Intraoperative ultrasonography has proven to be extremely beneficial and some surgeons argue that abdominal exploration combined with intraoperative ultrasonography alone may be useful [16]. However preoperative localization has its benefits including planning the surgical approach in minimally invasive surgery [15], and avoiding the risk of lengthy and extensive surgery [9]. As done in this case, EUS-FNT can be safely used to aid in the localization of small tumours especially for planned laparoscopic enucleations or resections, where intraoperative palpation cannot be done [17,18]. ^68^Ga-DOTATATE PET/CT has been shown to be a useful non-invasive adjunct imaging study capable of identifying most insulinomas, and may be considered when all imaging studies are negative and when a minimally invasive surgical approach is planned [19].

Surgical cure rates for insulinoma approach 100 % [7]. Options for surgery include enucleation, when technically feasible, or segmental resection of the pancreas, distal pancreatectomy or pacreaticoduodenectomy done for lesions involving a large portion of the pancreas, located deep within the pancreatic head in close proximity to the duct, or deep within the pancreatic tail due to a higher risk of duct injury and fistula formation after enucleation [2,15]. Postoperative complications include pancreatic fistula, pseudocyst, pancreatitis, diabetes, intra-abdominal abscess and haemorrhage. Fistulas are the most frequent complication and can be successfully managed conservatively with drainage, parenteral nutrition and somatostatin analogues [2,15].

## 4 Conclusion

Insulinomas can present as mimics of neurological and psychiatric conditions, leading to delayed diagnosis and prolonged morbidity. Most of these patients, like in this case report, receive unnecessary escalations in pharmacotherapy. A carefully taken history is the primary tool in the reassessment of patients with unusual and refractory neuropsychiatric conditions. Patterns of symptom occurrence after periods of fasting or caloric deficit usually become obvious in most patients with insulinoma. Surgical resection is curative and restores quality of life to pre-illness level with minimal complications.

## Ethical approval

Ethical approval was not required for this case report in compliance with the institutional regulations set forth by the University Hospital Frankfurt, where the majority of the patient's care and medical records were obtained, and as per German and Ghanaian national guidelines for reporting de-identified individual cases.

## CRediT authorship contribution statement

Emmanuel Agbozo: clinical diagnosis of patient; preparation, writing, reviewing and editing of the manuscript.

## Guarantor

Emmanuel Agbozo.

## Informed consent

Written informed consent was obtained from the patient for publication of this case report and associated images. A copy of the written consent is available for review by the Editor-in-Chief of this journal on request.

## Funding

The author received no funding or financial support for the research, authorship and/or publication of this article.

## Declaration of competing interest

Nothing to declare.
